# IE1 of Human Cytomegalovirus Inhibits Necroptotic Cell Death via Direct and Indirect Modulation of the Necrosome Complex

**DOI:** 10.3390/v16020290

**Published:** 2024-02-13

**Authors:** Anna Theresa Heusel, Sophie Rapp, Thomas Stamminger, Myriam Scherer

**Affiliations:** Institute of Virology, Ulm University Medical Center, 89081 Ulm, Germany; anna.heusel@uni-ulm.de (A.T.H.); sophie.rapp@hochschule-bc.de (S.R.)

**Keywords:** necroptosis, necroptotic cell death, HCMV, cytomegalovirus, IE1, RIPK3, MLKL, intrinsic immunity, innate immunity, interferon signaling

## Abstract

Programmed necrosis is an integral part of intrinsic immunity, serving to combat invading pathogens and restricting viral dissemination. The orchestration of necroptosis relies on a precise interplay within the necrosome complex, which consists of RIPK1, RIPK3 and MLKL. Human cytomegalovirus (HCMV) has been found to counteract the execution of necroptosis during infection. In this study, we identify the immediate-early 1 (IE1) protein as a key antagonist of necroptosis during HCMV infection. Infection data obtained in a necroptosis-sensitive cell culture system revealed a robust regulation of post-translational modifications (PTMs) of the necrosome complex as well as the importance of IE1 expression for an effective counteraction of necroptosis. Interaction analyses unveiled an association of IE1 and RIPK3, which occurs in an RHIM-domain independent manner. We propose that this interaction manipulates the PTMs of RIPK3 by promoting its ubiquitination. Furthermore, IE1 was found to exert an indirect activity by modulating the levels of MLKL via antagonizing its interferon-mediated upregulation. Overall, we claim that IE1 performs a broad modulation of innate immune signaling to impede the execution of necroptotic cell death, thereby generating a favorable environment for efficient viral replication.

## 1. Introduction

During viral infections, multiple sensing and signaling pathways need to be initiated and executed in a precise interplay to limit viral dissemination. The activation of programmed cell death pathways is of fundamental importance for intrinsic immune defenses as it allows for the rapid elimination of infected cells. The mechanisms of intrinsic immunity, such as programmed cell death or restriction factors, provide an immediate response to invading pathogens, thus representing one of the first lines of host defense. Besides the well-known cell death pathway apoptosis, an alternative form of programmed cell death was characterized in recent decades and termed as necroptosis [1]. In contrast to apoptosis, which results in cell shrinkage, membrane blebbing and apoptotic body formation, necroptotic cells are characterized by organelle swelling, leading to membrane swelling and subsequent membrane rupture [2,3].

Necroptosis can be triggered by numerous stimuli including the ligation of Toll-like, interferon (IFN) and tumor necrosis factor (TNF) receptors [4,5,6]. Within the well-studied TNF signaling pathway, the receptor-interacting protein kinase 1 (RIPK1 or RIP1) represents the switching point for the cellular fate, as the polyubiquitination of RIPK1 initiates cell survival pathways, whereas the deubiquitination of RIPK1 leads to the initiation of cell death [7]. Upon the deubiquitination of RIPK1, apoptotic cell death is initiated under control of the pro-apoptotic enzyme caspase-8 [8]. When caspase-8 is not active, due to genetic ablation or viral inhibition, the death model switches from apoptosis to necroptosis. Therefore, necroptosis has been described as back up mechanism for inhibited or limited apoptosis [9]. For the execution of necroptotic cell death, a close interplay between three key components is required, which form the so-called necrosome. Besides RIPK1, the necrosome complex is composed of the receptor-interacting protein kinase 3 (RIPK3 or RIP3) as well as the mixed lineage kinase domain like pseudokinase (MLKL) [10]. RIPK1 and RIPK3 share high structural similarities as both proteins encode for an N-terminal kinase domain but RIPK3 only encodes for a short C-terminal region with two disordered domains, whereas RIPK1 exhibits an additional intermediate domain and a C-terminal death domain [11]. During necroptotic signaling, the necrosome proteins interact with each other in a cascadic manner and undergo post-translational modifications (PTMs): RIPK1 interacts with RIPK3 via specific RIP homotypic interaction motifs (RHIM), which is accompanied by mutual phosphorylation events [12,13]. Phosphorylated and thereby activated RIPK3 conducts the phosphorylation of MLKL, leading to the formation of MLKL oligomers and the subsequent translocation to the plasma membrane [14]. The integration of these oligomers into the plasma membrane and the subsequent loss of membrane integrity ultimately lead to cell death [15,16]. In addition to multiple phosphorylation events, modifications with ubiquitin molecules have been proposed as an integral pillar for necroptotic signaling. In addition to the already mentioned ubiquitination of RIPK1, several ubiquitination sites within RIPK3 have been identified and reported to act either positively or negatively on necroptotic signaling [17,18,19].

The tightly regulated interactions and post-translational modifications within the necrosome complex represent the prerequisite of an efficient execution of necroptosis. Therefore, it is not surprising that many viruses induce the disruption of this complex in order to prevent necroptotic cell death and ensure the efficient production of viral progeny. Especially members of the highly host-adapted herpesvirus family have been shown to counteract necroptosis by the expression of distinct viral inhibitors that comprise RHIM domains and can therefore directly interfere with necrosome integrity. This strategy has been described for herpes simplex viruses 1 and 2, by the expression of the viral inhibitors ICP6 and ICP10, respectively, as well as for murine cytomegalovirus (MCMV) by the expression of the viral inhibitor of RIP kinase activation (vIRA) [20,21].

Another herpesvirus which has been reported to inhibit necroptosis is the human betaherpesvirus 5 or human cytomegalovirus (HCMV). HCMV is a widespread human pathogen with a high seroprevalence in the adult population [22]. Whereas infections with HCMV usually proceed asymptomatically, infections can cause severe or even life-threatening diseases in immunocompromised patients [23]. As a member of the beta-subgroup of herpesviruses, HCMV is characterized by a long replication cycle (72–96 h) and therefore depends on prolonged cell viability. However, it does not appear to encode any RHIM-containing proteins [24]. Instead, Fletcher-Etherington et al. postulated that the viral protein pUL36 is involved in the antagonism of necroptosis during HCMV infection, by initiating the degradation of the MLKL protein [25]. However, this activity of pUL36 appears to be strain specific as it has been observed for the genetically restored HCMV strain Merlin but not for the laboratory-adapted strain AD169.

Another study by Omoto et al. indicated that the presence of the immediate-early 1 (IE1) protein during HCMV infection is required for the inhibition of TNF-induced necroptosis [24]. IE1 is already expressed 2 h post infection (hpi) [26] and comprises four major structural domains: a disordered N-terminus, a globular core domain, a disordered C-terminus and a chromatin tethering domain (CTD) [27,28]. Furthermore, IE1 has been described to be post-translationally modified via phosphorylation and SUMOylation in order to achieve full functionality [28,29]. The early expression of IE1 predestines the protein as antagonist of early immune mechanisms. Indeed, IE1 has been identified as antagonist of intrinsic immunity, since it disrupts subnuclear structures known as promyelocytic leukemia nuclear bodies (PML-NBs) which inhibit the onset of HCMV gene expression [30]. Furthermore, IE1 directly interferes with type I interferon (IFN) signaling via binding signal transducers and activation of transcription (STAT) proteins thereby blocking the upregulation of antiviral IFN-stimulated genes (ISGs) [31,32]. While these activities have been shown to contribute to the success of lytic HCMV replication, a potential role of IE1 in the modulation of necroptosis remains to be elucidated.

In this study, we aimed at a detailed analysis of necroptotic signaling during HCMV infection and the identification of viral strategies to inhibit the execution of necroptosis. Our data show that HCMV infection leads to a strong modulation of necrosome proteins, particularly to a massive upregulation of post-translational modifications. We further found that HCMV blocks the execution of necroptotic cell death already at the early stages of infection and we identified the IE1 protein as potent antagonist of necroptotic cell death. Importantly, our data indicate a dual anti-necroptotic activity of IE1 since we observed a direct interaction with RIPK3 on one hand and an indirect modulation of MLKL levels via the antagonism of the IFN pathway on the other hand. Finally, we provide evidence that IE1 induces a broad modulation of genes involved in necroptotic signaling in order to establish a beneficial cellular environment for productive viral replication.

## 2. Materials and Methods

### 2.1. Oligonucleotides, Plasmids and Cloning

The oligonucleotides used for cloning were purchased from Biomers GmbH (Ulm, Germany). For the IFN-experiments in RT-qPCR, the oligonucleotides for MLKL were designed according to [33] and also purchased from Biomers GmbH (Ulm, Germany). The sequences of all Biomers Primers are listed in Table S1. For detection of GAPDH the Real Time PCR Primer Set VHPS-3541 was used (Biomol GmbH, Hamburg, Germany). To generate a construct with stable RIPK3 expression (pHM4730), RIPK3 was cloned from hRIP3 GFP WT (a gift from Francis Chan (Addgene plasmid #41387; http://n2t.net/addgene:41387 (accessed on 22 December 2023); RRID:Addgene_41387) [12]) into an already described pLKO-based lentiviral backbone (pHM4292, [34]) using PacI and SalI. For transfection experiments, RIPK3-MYC (pHM5136) and truncated FLAG-tagged RIPK3 variants were generated (pHM5304-5306, pHM5324). pHM5136 was generated by amplifying RIPK3 from pHM4730 and cloning into pcDNA3 backbone (Thermo Fisher Scientific Inc., Waltham, MA, USA) by using HindIII and XhoI. Truncated FLAG-tagged RIPK3 variants were cloned from pHM5136 by using Bsu15I and XhoI into a FLAG-pcDNA3 backbone (pHM971, described in [35]. The FLAG-MLKL construct (pHM5117) was cloned from hMLKL-Venus (a gift from Douglas Green (Addgene plasmid #106078; http://n2t.net/addgene:106078 (accessed on 22 December 2023); RRID:Addgene_106078) [36]) into pHM4292 using PacI and XbaI. IE1 in a pcDNA3 backbone (pHM494) was generated as described elsewhere [35]. For the generation of FLAG-IE1 aa1-491 (pHM5156) and FLAG-IE1 aa1-382 (pHM5153), IE1 was amplified from pHM494 and cloned into pHM971 by using BamHI and XbaI. FLAG-FEN1 aa175-366 (pHM4273) was generated as described elsewhere [37]. For IE1 purification, codon-optimized IE1 in a pGEX-6P-1 backbone was generated as described elsewhere [27].

### 2.2. Cultivation of Cells and Generation of RIPK3 Expressing Cells

Primary human foreskin fibroblasts (HFF) and HEK293T cells were cultivated in Dulbecco’s minimal essential medium (DMEM, Gibco, Thermo Fisher Scientific Inc., Waltham, MA, USA), supplemented with 10% fetal bovine serum (FBS, Sigma-Aldrich, Merck KGaA, Darmstadt, Germany) and 1% penicillin-streptomycin (Pen/Strep, Sigma-Aldrich, Merck KGaA, Darmstadt, Germany). HFF with an inducible IE1 or IE1 aa1-382 expression (generated as described elsewhere [34]) were cultivated with 10% tetracycline-free FBS (Clontech, Takara, Saint-Germain-enLaye, France) and 1% Pen/Strep. Gene expression of inducible cell lines was stimulated for 24 h with doxycycline (0.5 µg/mL, Sigma-Aldrich, Merck KGaA, Darmstadt, Germany). HFF cells were additionally cultivated in presence of 500 µg/mL geneticin (InvivoGen, Toulouse, France). HEC-LTT cells (immortalized HUVEC cell line [38]) were kindly provided by C. Sinzger (Ulm, Germany) and cultivated as described elsewhere [39]. Expressing RIPK3 cells were generated via lentiviral-mediated gene transfer according to Schweininger et al., 2021 [40]. For the generation of lentiviral particles, the expression plasmid pHM4730 was used and RIPK3-positive cell populations were selected by using geneticin (500 µg/mL).

### 2.3. Treatment of Cells and Cell Viability Assay

The indicated HFF cells were stimulated with interferons (IFNs) for 24 h by applying 1000 U/mL of IFN-α, IFN-β or IFN-γ (all purchased from R&D systems, Minneapolis, MN, USA). For the TBZ treatment, the indicated HFF cells were stimulated by using a combination of TNF-α (30 ng/mL, R&D systems, Minneapolis, MN, USA), BV-6 (5 µM) and z-VAD-fmk (25 µM, both purchased from Selleck Chemicals LLC, Houston, TX, USA) for up to 8 h. As a negative control, the indicated cells were treated with DMSO (Sigma-Aldrich, Merck KGaA, Darmstadt, Germany). To investigate ubiquitination, cells were treated for 6 h with the ubiquitination inhibitor PYR-41 (5 µM, Selleck Chemicals LLC, Houston, TX, USA). Cell viability was quantified according to intracellular ATP levels by utilizing the CellTiter-Glo^®^ Luminescent Cell Viability Assay (Promega, Fitchburg, MA, USA) according to the manufacturer’s instructions and the luminescence was detected using a Hidex Chameleon platereader (Hidex, Mainz, Germany).

### 2.4. Infection Experiments and Generation of HCMV Deletion Mutants

Infection experiments were conducted by using the HCMV strains AD169 WT (HB15, [41]), AD169ΔIE1 (based on HB15), TB40/E WT (TB40/E-BAC4, [42]) or TB40/EΔIE1 (based on TB40/E-BAC4). Deletion strains were generated according to Tischer et al., 2006 [43]. For this technique, a recombination fragment was generated by using firstly the primer pair 57–76 and 57–77, and in a second round, the primer pair 57–75 and 57–78 (see sequences in Table S1). The template pEP-kanS was kindly provided by N. Osterrieder, Berlin, Germany. The PCR-product was subjected to DpnI digestion (Thermo Fisher Scientific Inc., Waltham, MA, USA) and gel purification using the GeneJET Gel Extraction Kit (Thermo Fisher Scientific Inc., Waltham, MA, USA). Subsequently, the recombination fragment was transformed into *Escherichia coli* strain GS1783 (a gift of M. Mach, Erlangen) already harboring HB15 or TB40/E-BAC4, and two-step bacteriophage red-mediated recombination was performed as described elsewhere [43]. Positive clones were identified by selection for kanamycin (first recombination) or chloramphenicol and 1% arabinose (second recombination, all purchased from Sigma-Aldrich, Merck KgaA, Darmstadt, Germany). After BAC DNA isolation, successful recombination was confirmed by PCR, restriction digestion (HindIII, Thermo Fisher Scientific Inc., Waltham, MA, USA) and sequencing (sequences of used oligonucleotides see Table S1).

Generation of viral stocks and subsequent titration was performed as described in Scherer et al., 2016 [44]. To ensure similar infection rates between WT and ΔIE1 HCMV strains, intracellular HCMV genome equivalents were determined 8 hpi, according to corresponding levels of gB as described elsewhere [44].

For experiments with UV-inactivated virus, viral dilutions were UV-treated for 2 min with 0.12 J/cm² using CL-1000 Ultraviolett Crosslinker (UVP, Analytik Jena GmbH & Co KG, Jena, Germany.

### 2.5. Transfection and Co-Immunoprecipitation

Transfection and co-immunoprecipitation (Co-IP) experiments were performed either in HEK293T or in HFF cells (control or RIPK3) as described elsewhere [45] with the modification that no sonification of the lysates was performed. For precipitation, either protein A-sepharose beads (Sigma-Aldrich, Merck KgaA, Darmstadt, Germany) coupled to anti-FLAG antibody (M2, Sigma-Aldrich, Merck KgaA, Darmstadt, Germany) or anti-IE1 [46] were used or MCE anti-FLAG magnetic beads (MedChemExpress, Monmouth Junction, NJ, USA) were utilized. For the Co-IPs with truncated RIPK3 variants, purified IE1 was utilized. For that, IE1 was expressed in bacteria and subsequently purified according to Scherer et al., 2014 [27].

### 2.6. Western Blot, Indirect Immunofluorescence and Antibodies

For Western blot analysis whole cell lysates were prepared by removing cellular debris by centrifugation and boiling the cells at 95 °C for 10 min with 1× ROTI-Load 1 (Carl Roth, Karlsruhe, Germany). Afterwards, the samples were sonicated with an amplitude of 100% for 60–90 s by using Q700 sonicator (Qsonica, Newton, MA, USA). Next, the samples were subjected to SDS-PAGE by using 8 or 12% SDS-polyacrylamide gels. Subsequently, the samples were transferred onto PVDF membranes (Bio-Rad, Feldkirchen, Germany) followed by chemiluminescence-mediated detection of proteins. Imaging was done by using a Fusion FX7 Spectra (Vilber Lourmat, Eberhardzell, Germany). For quantification, relative signal intensities were determined by using the software Image Lab 6.0 (Bio-Rad, Feldkirchen, Germany) and normalized to levels of β-actin.

For indirect immunofluorescence, samples were prepared as described by Rothemund et al., 2022 [45].

The following antibodies were utilized for the analyses: anti-RIPK3 (mAb, E1Z1D), anti-RIPK1 (mAb, D94C12), anti-p-RIPK1 S166 (mAb, D1L3S), anti-MLKL (mAb, D2I6N) (all purchased from Cell Signaling Technology, Frankfurt am Main, Germany); anti-p-RIPK3 S227 (mAb, EPR9627) (purchased from Abcam, Berlin, Germany); anti-β-actin (mAb, AC-15), anti-FLAG (mAb, M2) (all purchased from Sigma-Aldrich, Merck KgaA, Darmstadt, Germany); anti-IE1 (p63-27) [46]; anti-CH443 (for IE1 aa1-382 detection, sc-69747, Santa Cruz Biotechnology, Dallas, TX, USA) anti-MCP (28-4) [47]; anti-ppUL44 (kindly provided by B. Plachter, Mainz, Germany); anti-pp65 (28-77, kindly provided by W. Britt, Birmingham, AL, USA, [47]); as secondary antibodies for Western blotting horseradish peroxidase (HRP)-conjugated goat anti-mouse/-rabbit antibodies were utilized (pAb, IgG H+L, Dianova, Hamburg, Germany); as a secondary antibody for indirect immunofluorescence an Alexa Fluor 488-conjugated goat anti-rabbit antibody (Thermo Fisher Scientific Inc., Waltham, MA, USA) was used.

### 2.7. RNA Isolation, Quantitative Reverse Transcription PCR (RT-qPCR) and Necroptosis-Related Profiling

Total RNA was isolated from non-infected and infected HFF cells according to Stilp et al., 2022 [48]. For the IFN-experiments, cDNA synthesis was performed using the Maxima First Strand cDNA synthesis kit from Thermo Fisher Scientific Inc. (Waltham, MA, USA) and the cDNA was supplied with the SsoAdvanced Universal SYBR Green Supermix (Bio-Rad, Feldkirchen, Germany), according to the manufacturer’s instructions, respectively. Primer pairs of GAPDH (Biomol, Hamburg Germany) and MLKL (Biomers GmbH, Ulm, Germany, according to Sun et al., 2019 [33]) were used for the investigation. RT-qPCR was conducted using the AriaMx Real-Time PCR System (Agilent, Santa Clara, CA, USA) according to Stilp et al., 2022 [48]. Data analysis was accomplished by using the AriaMx Software v1.5. For infection experiments, the RT² Profiler^TM^ PCR Array Human Necrosis was performed by using the cDNA synthesis kit RT² First Strand Kit and the RT² SYBR^®^ Green ROX qPCR Mastermix (all purchased from Qiagen, Düsseldorf, Germany), according to the manufacturer’s instructions, respectively. RT-qPCR was conducted using the QuantStudio^TM^ 3 Real-Time PCR System (Thermo Fisher Scientific Inc., Waltham, MA, USA) with thermal cycling conditions consisting of an initial denaturation step (10 min at 95 °C) followed by 40 amplification cycles (15 s at 95 °C, 60 s at 60 °C) and a final dissociation stage. Data analysis was accomplished by using the QuantStudio^TM^ Design and Analysis Software (v1.5.1). For data acquisition, mRNA levels were normalized to levels of housekeeping genes (*GAPDH* for IFN-experiments and *ACTB*, *B2M*, *HPRT1* and *RPLP0* for the profiler PCR array) and relative mRNA levels were quantified using the 2^−ΔΔCq^ method [49].

## 3. Results

### 3.1. Establishment of a Necroptosis-Sensitive Cell Culture Model

**Figure 1:**
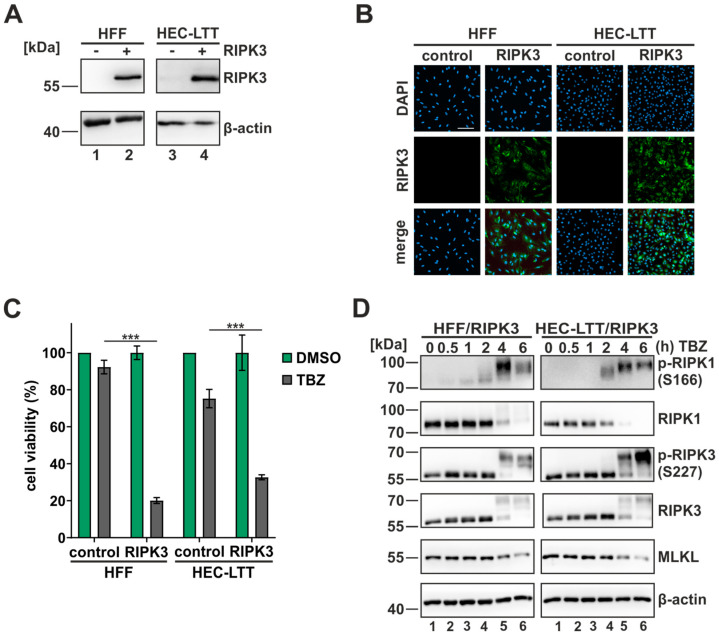
Generation of a necroptosis-sensitive cell culture model. (**A**) HFF and HEC-LTT cells were lentivirally transduced to stably express RIPK3. Successful integration of RIPK3 was monitored in Western blot analysis. (**B**) Expression of RIPK3 was analyzed by indirect immunofluorescence analysis. Cell nuclei were stained with DAPI. Scale bar, 100 µm. (**C**) The sensitivity of RIPK3 expressing cells to necroptosis was analyzed in a cell viability assay by monitoring intracellular ATP levels (CellTiter-Glo, Promega, Fitchburg, MA, USA). The cells were stimulated for 8 h with TBZ (TNFα (30 ng/mL), BV-6 (5 µM) and z-VAD-fmk (25 µM)). Green, DMSO-treated cells; grey, TBZ treated cells. Depicted values represent the means +/− SD derived from triplicates relative to control cells (%). (**D**) The modulation of the necrosome components after the indicated times of TBZ treatment was monitored in Western blot analysis. Β-actin served as internal loading control. Each experiment was performed three times in independent experiments and one representative experiment is shown. For statistical analysis a student’s *t*-test was performed (unpaired, two-tailed); *** *p* < 0.001.

To execute necroptotic cell death, a sophisticated interplay between the necrosome components RIPK1, RIPK3 and MLKL is required. During cell propagation, cells tend to lose the ability to actively express RIPK3 due to a transcriptional shutdown via epigenetic silencing [50]. To investigate necroptotic cell death in the context of HCMV infection, we first analyzed two HCMV permissive cell types for RIPK3 expression. In primary human fibroblasts (HFF) and conditionally immortalized human umbilical vein endothelial cells (HEC-LTT), no RIPK3 expression was detected in Western blot analysis. As previously shown, RIPK3 can be reintroduced via lentiviral-mediated gene transfer to enable an analysis of necroptosis [24]. Therefore, HFF and HEC-LTT cells were lentivirally transduced and subsequently selected for geneticin-resistant cell populations, which yielded high transduction efficiencies about 90%. The transduction was monitored through Western blot and indirect immunofluorescence (Figure 1A,B). To verify the necroptosis-sensitivity of the generated RIPK3 cells, the cells were subjected to TBZ treatment, which induces TNF-dependent necroptosis [24]. The TBZ treatment is a combination of TNFα (30 ng/mL, cytokine) to induce the signaling pathway, BV-6 (5 µM, SMAC mimetic) to inhibit cell survival pathways and z-VAD-fmk (25 µM, pan-caspase inhibitor) to switch the death pattern from apoptosis to necroptosis. For both cell types, a strong reduction of cell viability to approx. 70–80% in the presence of RIPK3 was observed (Figure 1C), thus confirming the sensitivity to necroptosis. Without RIPK3 expression, an only marginally reduced cell viability of approx. 10% was detected in HFF, compared to an approx. 25% reduction in HEC-LTT. This difference may be attributed to cell type specificities, indicating that HEC-LTT cells are more susceptible for RIPK3-independent pathways than HFF cells. To monitor the necrosome complex upon necroptosis induction, both cell types were treated with TBZ up to 6 h and cell lysates were analyzed in Western blot experiments. The necrosome components displayed a strong modulation upon necroptosis induction for both cell types (Figure 1D). Especially for RIPK3 and RIPK1, strong upshifts were observed which were most clearly detected with phosphorylation-specific antibodies recognizing necroptosis-specific phosphorylation sites of RIPK1 (S166) and RIPK3 (S227) [14,51]. Intriguingly, the modification of RIPK3 in HEC-LTT/RIPK3 cells was stronger compared to HFF/RIPK3 cells whereas for RIPK1 the opposite effect was observed. These differences indicate cell type specific responses to the TBZ treatment. Taken together, by restoring RIPK3 expression, we established a cell culture model in which necroptotic signaling can be studied in the context of HCMV infection.

### 3.2. Strong Modulation of Necrosome Components during HCMV Infection 

**Figure 2:**
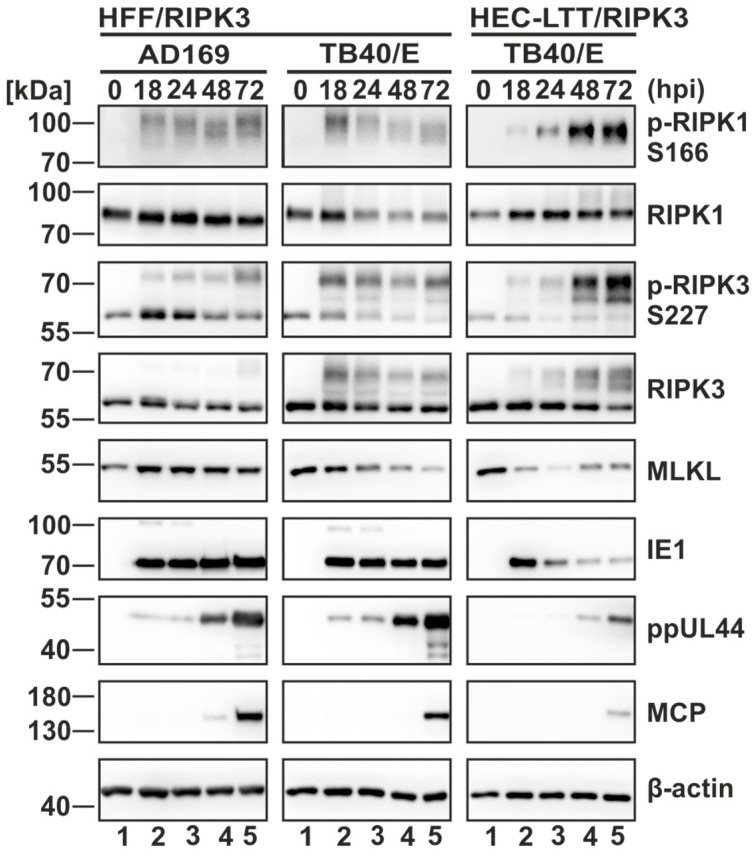
Necrosome components are strongly modulated during HCMV infection. RIPK3 expressing HFF and HEC-LTT cells were infected for the indicated times and then harvested for Western blot analysis. HFF/RIPK3 cells were infected with AD169 (left panel) or TB40/E (middle panel) and HEC-LTT/RIPK3 with TB40/E (right panel). Infections were performed at an MOI of 5. Necrosome components and viral markers of infection were analyzed by Western blot experiments. Β-actin served as internal loading control. Three independent experiments were performed, and one representative experiment is shown.

After establishing necroptosis-sensitive cells, we analyzed the effect of HCMV infection on the individual necrosome components. For this, RIPK3 expressing HFF and HEC-LTT were infected with the high-passage HCMV strain AD169 or the clinical-like HCMV strain TB40/E at a multiplicity of infection (MOI) of 5. At 18–72 h post infection (hpi), the cells were harvested and subjected to Western blot analysis (Figure 2). Successful infection was monitored by staining viral marker proteins for the immediate-early (IE1), early (ppUL44) and late (MCP) phase of viral replication. Notably, both HCMV strains induced a robust modulation of the necrosome components in HFF. However, while TB40/E appeared to strongly modulate the necrosome already at 18 hpi, the effect continuously increased until 72 hpi in AD169 infected cells. Regarding the modulation of MLKL, a significant difference between AD169- and TB40/E-infected cells became evident. Whereas TB40/E-infected cells exhibited a substantial reduction of MLKL levels starting at 24 hpi, AD169-infected cells displayed an upregulation of MLKL levels beginning at 18 hpi. These findings align with the data of Fletcher-Etherington et al. demonstrating that a degradation of MLKL occurs after infection with the HCMV strain Merlin, whereas AD169 is incapable of initiating MLKL degradation due to a point-mutation in the effector protein pUL36 [25]. In HEC-LTT/RIPK3 cells, we observed a similar modulation of the necrosome components after infection with TB40/E, however, the kinetics appeared to be different compared to TB40/E-infected HFF cells. This indicates cell type-specific differences regarding alterations of the necrosome complex during HCMV infection. Although the cells were infected for 3 days, representing a full replication cycle of HCMV, and strong modifications of the necrosome complex were detected, no notable reduction of the levels of the housekeeping gene β-actin occurred. In conclusion, these data suggest that HCMV induces an overall upregulation of necrosome modifications independent of cell type and virus strain.

### 3.3. Counteraction of Necroptosis during HCMV Infection at Early Stages of Infection 

**Figure 3:**
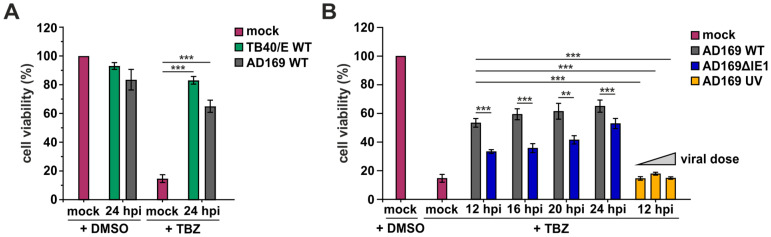
HCMV-mediated rescue from necroptotic cell death begins already at the early stages of infection. HFF/RIPK3 cells were infected for the indicated times and subsequently treated with TBZ for 8 h or with DMSO as control. Cells were infected with TB40/E WT (**A**), AD169 WT (**A**,**B**) and AD169 ΔIE1 (**B**) at an MOI of 3. AD169 UV (**B**) was used at increasing viral doses (calculated MOIs of 6, 12 and 18). Necroptotic cell death was monitored by analyzing intracellular ATP levels using a cell viability assay (CellTiter-Glo, Promega, Fitchburg, MA, USA). Red, mock infected cells; green, TB40/E WT infected cells; grey, AD169 WT infected cells; blue, AD169ΔIE1 infected cells; yellow, AD169 UV infected cells. Depicted values (%) represent the mean +/− SD derived from triplicates relative to the control (mock, DMSO). Each experiment was performed two times in independent experiments and one representative experiment is shown. For statistical analysis a student’s *t*-test was performed (unpaired, two-tailed); ** *p* < 0.01, *** *p* < 0.001.

To further elucidate the anti-necroptotic activity of HCMV, the cell viability of HFF/RIPK3 cells was monitored during HCMV infection. For this purpose, HFF/RIPK3 cells were infected at an MOI of 3 with the HCMV strains TB40/E and AD169 and necroptosis was induced at 24 hpi via TBZ treatment. Cells that were infected prior to TBZ treatment exhibited significantly higher cell viability compared to cells which were not infected prior to TBZ treatment, indicating an HCMV-induced resistance to necroptotic cell death (Figure 3A). This rescue effect appeared to be slightly more efficient after infection with TB40/E compared to AD169. This finding is particularly interesting, considering the postulation that AD169 expresses a non-functional pUL36, which is no longer able to degrade MLKL and antagonize necroptosis during HCMV infection [25]. Thus, our data indicate that another HCMV protein besides pUL36 exhibits strong anti-necroptotic activity. Next, we wanted to narrow down the time schedule of the antagonism of necroptosis during HCMV infection. Therefore, we infected HFF/RIPK3 cells with AD169 at an MOI of 3 for 12–24 h, subsequently conducted TBZ treatment and monitored the cell viability (Figure 3B). It became evident that the counteraction of necroptosis occurred at the early stages of infection, as already 12 hpi a higher cell viability was achieved in AD169 wildtype (WT) infected cells compared to non-infected cells. Overall, the cell viability increased with an increasing time of infection prior to TBZ treatment. This tendency suggests that an immediate-early gene product of HCMV is crucial for the antagonism of necroptosis during HCMV infection, aligning with data from Omoto and colleagues [24]. To address this hypothesis, we included an HCMV strain with a deletion of exon 4 of the major immediate-early gene region in our analysis, which is defective for IE1 expression (ΔIE1). For AD169ΔIE1, we observed a significantly reduced cell viability compared to AD169 WT indicating that IE1 either directly or indirectly contributes to the inhibition of necroptosis (Figure 3B). Since infection with AD169ΔIE1 still inhibited necroptosis to some extent, we wondered whether viral tegument proteins could contribute to the inhibition of necroptotic cell death at the early stages of infection. Therefore, we included UV-inactivated AD169 (AD169 UV) in our analysis (Figure 3B). Successful UV-inactivation was monitored by staining IE1 and the tegument protein pp65 in Western blot analysis (Figure S1). In cells infected with AD169 UV, no rescue effect on necroptosis induction via TBZ treatment was observed. This observation held true even with higher viral doses, indicating that tegument proteins tend not to play a role in antagonizing necroptotic cell death during early stages of infection. 

In summary, we could demonstrate that necroptotic cell death is antagonized during HCMV infection even in absence of the so far identified necroptosis inhibitor pUL36. The antagonism of necroptosis occurs already at the early stages of infection and appears to be dependent on the expression of IE1.

### 3.4. HCMV Effector Protein IE1 Affects Necroptotic Signaling by Promoting Ubiquitination of RIPK3 

**Figure 4:**
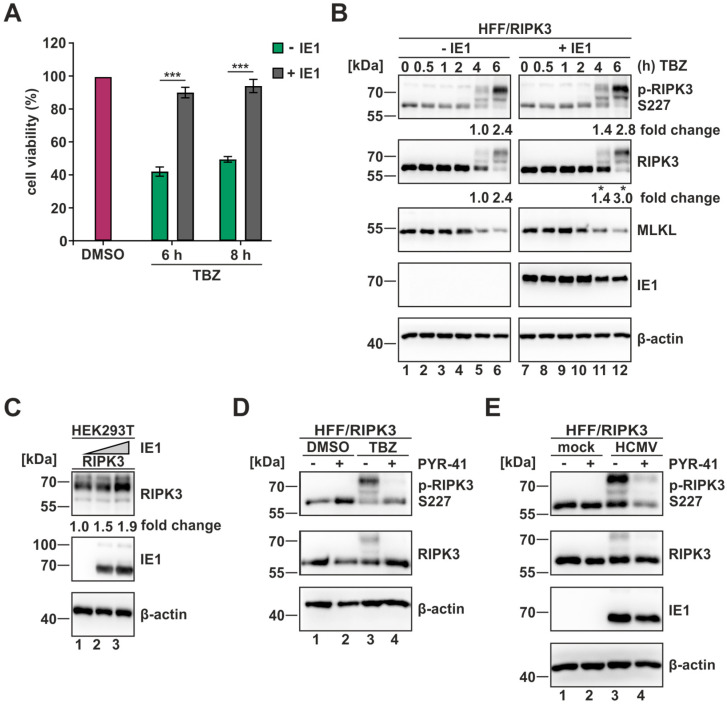
The immediate-early 1 (IE1) protein exhibits a strong anti-necroptotic activity by modulating the modification pattern of RIPK3. (**A**) HFF/RIPK3 cells with inducible IE1 expression (**A**,**B**) were treated with TBZ for the indicated times or with DMSO as control. Red, DMSO-treated cells; green, no IE1 expression; grey, IE1 expression. Depicted values represent the mean +/− SD derived from triplicates relative to the DMSO control (%). (**B**) Expression levels of p-RIPK3 S227, RIPK3 and MLKL were analyzed in presence or absence of IE1 after the indicated times of TBZ treatment by Western blot analysis. – IE1, no IE1 expression, + IE1, IE1 expression. (**C**) Expression plasmids of IE1 and RIPK3 were co-transfected in HEK293T cells, whereby increasing amounts of IE1 were used. The expression levels were monitored in Western blot analysis. (**D**) To confirm a ubiquitination of RIPK3 during necroptosis induction, HFF/RIPK3 cells were treated with DMSO or TBZ for 6 h +/− PYR-41 (inhibitor of ubiquitination, Selleck Chemicals LLC, Houston, TX, USA). (**E**) To confirm the ubiquitination of RIPK3 during HCMV infection, HFF/RIPK3 cells were infected with TB40/E at an MOI of 3 and at 24 hpi, PYR-41 was applied for 6 h. Protein levels of RIPK3 and IE1 were monitored in Western blot analysis. β-actin served as internal loading control. Each experiment was performed at least two times in independent experiments and always one representative experiment is shown. Fold changes of protein expression were determined according to signal intensities normalized to β-actin levels. For statistical analysis a student’s *t*-test was performed (unpaired, two-tailed); * *p* < 0.05, *** *p* < 0.001.

Having shown that the effector protein IE1 is important in counteracting necroptosis during HCMV infection, we next wanted to investigate the impact of IE1 on necroptotic signaling in absence of other viral proteins. Thus, we generated HFF/RIPK3 cells with inducible IE1 expression, in which the expression of IE1 can be stimulated by treatment with doxycycline. To determine whether IE1 expression has a direct effect on the execution of necroptosis, the IE1-inducible cells were treated with TBZ for 6–8 h or DMSO as control, and necroptotic cell death was quantified using a cell viability assay (Figure 4A). While the cell viability was reduced to about 50–60% in cells without IE1 expression (− IE1), IE1 expressing cells were similar to DMSO treated cells (+ IE1). This evident effect highlights the anti-necroptotic activity of IE1, which is exerted independently of other viral proteins. To examine whether IE1 expression directly modulates the necroptotic cascade, the inducible IE1 cells were subjected to TBZ treatment for 0.5–6 h and the levels of RIPK3 and MLKL were examined via Western blot analysis (Figure 4B). For RIPK3, more pronounced modifications were observed in the presence of IE1 (+ IE1) at 6 h post TBZ treatment, while levels of MLKL appeared unaffected by IE1 expression. Simultaneously, a C-terminal deletion mutant of IE1 (IE1 aa1-382) was tested in a similar experimental setup. No altered modification pattern of RIPK3 was detected for this mutant (Figure S2), indicating that the impact on RIPK3 modification is only induced by the full-length version of IE1. Stronger modification of RIPK3 was additionally observed in co-transfection experiments in HEK293T cells, where increasing amounts of IE1 likewise led to an increased modification of RIPK3 (Figure 4C). The modified versions of RIPK3 can be also visualized with a phospho-RIPK3 antibody (Figure 4B), but the upshift of RIPK3 is comparatively strong to result only from phosphorylation events. To analyze whether these slowly migrating bands could be due to the ubiquitination of RIPK3, we included an inhibitor of ubiquitination (PYR-41) in our analysis. By using this inhibitor, the formation of high molecular weight isoforms of RIPK3 could be reduced, both in TBZ treated cells (Figure 4D) and in infected cells (Figure 4E). Since several publications report on the anti-necroptotic effect of RIPK3-ubiquitination [18,19], it appears likely that IE1 interferes with necroptotic signaling by promoting the ubiquitination of RIPK3. Taken together, our data indicate that IE1 exhibits strong anti-necroptotic activity and we provide evidence that IE1 manipulates the modification pattern of RIPK3 during necroptotic signaling.

### 3.5. Direct Interference of IE1 with the Necroptotic Cascade by Interacting with RIPK3 

**Figure 5:**
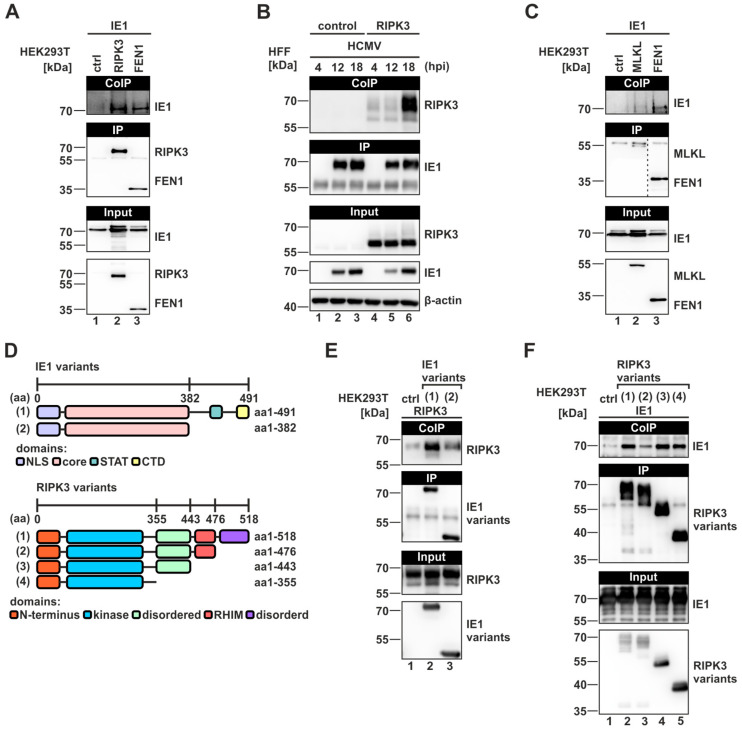
IE1 associates with the necrosome complex via binding RIPK3 and this interaction is promoted by distinct domains of IE1 and RIPK3. (**A**,**C**) HEK293T cells were co-transfected with expression plasmids for the indicated proteins and co-immunoprecipitation (Co-IP) was performed. FEN1-IE1 interaction served as positive control [37]. The IP of FLAG-tagged RIPK3, MLKL and FEN1 constructs was performed by using protein A-sepharose beads with immobilized anti-FLAG antibody. (**B**) HFF/control and HFF/RIPK3 were infected with TB40/E at an MOI of 1 for 4–18 h and subsequently IE1 was precipitated using protein A-sepharose beads with immobilized anti-IE1. (**D**) Schematic illustration of truncated variants of IE1 (upper panel) and RIPK3 (lower panel) which were used for fine-mapping of the interaction interface (**E**,**F**). NLS, nuclear localization sequence; core, globular core domain; STAT, binding domain of STAT proteins; CTD, chromatin tethering domain; RHIM, RIP homotypic interaction motif. (**E**,**F**) Fine-mapping of the interaction interface of IE1 and RIPK3 in HEK293T by transfecting expression plasmids encoding FLAG-tagged IE1 and RIPK3 variants and subsequent Co-IP analysis by using magnetic beads with immobilized anti-FLAG antibody. In (**F**), purified protein of IE1 was utilized. Each experiment was performed at least three times and one representative experiment is shown.

Having identified IE1 as an antagonist of necroptosis, we aimed to investigate whether IE1 directly interferes with the necrosome components to modulate necroptotic signaling. As we observed alterations in the modification pattern of RIPK3 induced by IE1, we first tested whether an interaction between these two proteins can be detected. For that, HEK293T cells were co-transfected with RIPK3 and IE1 constructs and subsequently a co-immunoprecipitation (Co-IP) was conducted. As a positive control, the previously described interaction between IE1 and the flap endonuclease 1 (FEN1) was utilized [37]. As depicted in Figure 5A, upper panel, both FEN1 and RIPK3 were able to co-precipitate IE1. This result suggests that IE1 interferes with early events of necroptotic signaling by interacting with RIPK3. To validate the interaction between RIPK3 and IE1 under infection conditions, HFF/control and HFF/RIPK3 cells were infected with TB40/E at an MOI of 1 for 4–18 h, followed by a Co-IP conducted by precipitating IE1. At 18 hpi, a strong interaction between IE1 and RIPK3 was detected (Figure 5B, upper panel). Some non-specific signals were observed at earlier times of infection. Intriguingly, the IE1-mediated precipitation resulted in the pulldown of modified RIPK3, further emphasizing our previous finding that IE1 promotes the modification of RIPK3. This interaction between IE1 and RIPK3 is particularly interesting, considering that HCMV was reported to lack RHIM-containing proteins [24], and thus far an RHIM-independent interaction with the necrosome has only been described for human gammaherpesvirus 4 (Epstein–Barr virus) [52]. To determine whether IE1 specifically binds to RIPK3, interaction analysis between MLKL and IE1 was performed, but here, no co-precipitation of IE1 could be detected (Figure 5C, upper panel). To further narrow down the interaction interface of IE1 and RIPK3, several truncated variants of these proteins were generated and analyzed in Co-IP experiments (schematic illustration in Figure 5D). We observed that the interaction with RIPK3 depends on the disordered C-terminal domain of IE1, since an IE1 variant lacking the C-terminus (IE1 aa1-382) showed strongly reduced binding to RIPK3 (Figure 5E, upper panel, lane 3). Next, we investigated whether post-translational modifications like SUMOylation or phosphorylation of IE1 are required for the interaction with RIPK3. For this, IE1 was expressed in bacteria and purified as described previously [27]. In Co-IP experiments using purified IE1, we still observed an interaction between RIPK3 and IE1 (Figure 5F, upper panel, lane 2), indicating that this interaction does not depend on IE1 modifications. By analyzing RIPK3 variants with a truncated C-terminus, interactions with IE1 could be detected, although to varying degrees (Figure 5F, upper panel, lanes 3–5). Intriguingly, a strong interaction between IE1 and the RIPK3 mutant aa1-443 (3) was detected. As neither this mutant nor IE1 contains an RHIM domain, these data suggest that the interaction of IE1 and RIPK3 is completely independent of RHIM domains. To sum up, our data provide evidence that IE1 can interfere with necroptotic signaling by binding RIPK3 in an RHIM-independent manner.

### 3.6. IE1 Inhibits the Interferon-Mediated Upregulation of MLKL 

**Figure 6:**
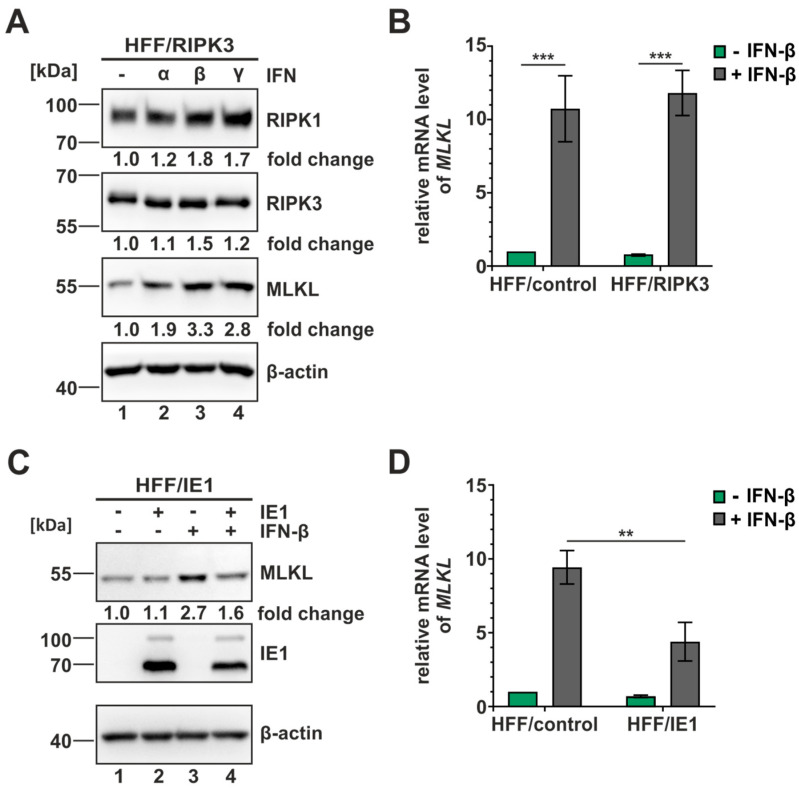
IE1 antagonizes the interferon-mediated upregulation of MLKL. HFF cells were treated with the indicated types of IFN (1000 U/mL) for 24 h. (**A**,**C**) Levels of necrosome components in the indicated HFF cells were analyzed in Western blot experiments. Β-actin served as internal loading control. Fold changes of protein expression were determined according to signal intensities normalized to β-actin levels. (**C**) − IE1, no IE1 expression; + IE1, IE1 expression; − IFN-β, no IFN-β stimulation; + IFN-β, IFN-β stimulation. (**B**,**D**) Modulation of MLKL transcription upon IFN-stimulation was analyzed by isolating total RNA and performing SYBR qPCR analysis in the indicated HFF cells. Depicted values represent the mean +/− SD derived from triplicates relative to untreated HFF/control cells and normalized to levels of the housekeeping gene *GAPDH*. Green, no IFN-β stimulation; grey, IFN-β stimulation. For statistical analysis a student’s *t*-test was performed with ΔCq-values (unpaired, two-tailed); ** *p* < 0.01, *** *p* < 0.001. Each experiment was performed three times in independent experiments and one representative experiment is shown.

Increasing evidence suggests a crucial role of interferon (IFN) signaling in the activation of necroptotic cell death. Several components of the necroptotic pathway, including MLKL, have been described to be upregulated by IFN treatment and generally, the IFN-homeostasis appears to be of high importance for the efficient execution of necroptosis [53,54]. Since IE1 is a known inhibitor of IFN signaling, it was tempting to speculate that this activity contributes to the inhibition of necroptosis in an indirect manner [32]. To address this hypothesis, we first analyzed whether we can detect the IFN-mediated upregulation of MLKL and other necrosome components in our necroptosis-sensitive cell system. Therefore, we stimulated the HFF/RIPK3 cells with different types of IFN (IFN-α, -β, -γ) for 24 h and subsequently harvested and analyzed the samples in Western blot (Figure 6A). Interestingly, we observed an upregulation of all necrosome components after the treatment with all three types of IFN. The strongest effect was detected for MLKL after IFN-β stimulation, with a fold change of 3.3. Next, we investigated whether this upregulation can also be detected on a transcriptional level. Therefore, total RNA was isolated after IFN-β stimulation (24 h), followed by quantification of the *MLKL* mRNA levels using RT-qPCR. In HFF/control as well as HFF/RIPK3, a significant upregulation of *MLKL* levels upon IFN-β stimulation was monitored (Figure 6B). After confirming the positive effect of IFN-β stimulation on MLKL levels, we examined the influence of IE1 expression in this context. For that, the inducible IE1 cells were utilized and stimulated with IFN-β for 24 h. Subsequently, the levels of MLKL were analyzed on protein and transcriptional levels by performing Western blot experiments and RT-qPCR, respectively (Figure 6C,D). In the presence of IE1, a diminished MLKL induction of approx. 50% was detected, both on the protein level (Figure 6C) and on transcriptional level (Figure 6D), indicating that IE1 is capable of antagonizing the IFN-mediated upregulation of MLKL. These observations suggest that IE1 plays a dual role in the antagonism of necroptotic cell death. On the one hand, it directly affects RIPK3, but on the other hand, it indirectly modulates the expression of MLKL.

### 3.7. Global Antagonism of IFN-Regulated Genes by IE1 to Limit the Execution of Necroptosis 

**Figure 7:**
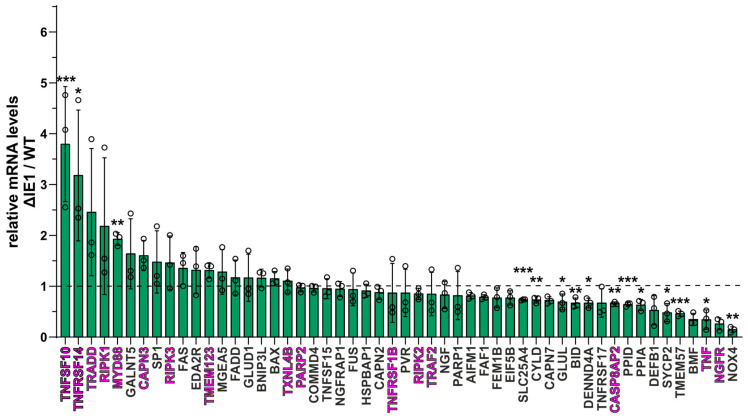
IE1 modulates the activation of innate immune signaling to circumvent necroptosis. HFF/RIPK3 cells were infected with WT and ΔIE1 HCMV (both TB40/E) for 24 h at an MOI of 3. Subsequently, total RNA was isolated and necrosis/necroptosis-related profiling was performed by using the RT² Profiler Necrosis PCR array (Qiagen, Düsseldorf, Germany). Values above the dashed line (at Y = 1) indicate an upregulation and values below the dashed line indicate a downregulation during ΔIE1 infection compared to WT infection. Genes illustrated in pink are described as ISGs [48]. Depicted values represent the mean +/− SD derived from three repetitions, ΔIE1 relative to WT infected HFFs. For normalization the mean Cq-value of the housekeeping genes *ACTB*, *B2M*, *HPRT1* and *RPLP0* was utilized. For statistical analysis a multicomparison *t*-test was performed with ΔCq-values (unpaired, two-tailed); * *p* < 0.05, ** *p* < 0.01, *** *p* < 0.001.

Given that fact several components of the necroptotic pathway have been described to be IFN-regulated [55,56], it was interesting to analyze whether IE1 affects a broader range of necroptotic key players in order to establish a favorable cellular environment for HCMV replication. To examine this hypothesis, total RNA from TB40/E WT- or ΔIE1-infected cells was analyzed using a Profiler PCR array (Qiagen, Düsseldorf, Germany), which allowed a diversified analysis of necrosis/necroptosis-related genes. After data acquisition, the levels of gene expression in ΔIE1-infected cells compared to WT-infected cells were calculated and depicted as a fold change in Figure 7. Interestingly, almost all genes included in this array appeared to be differentially regulated during infection with ΔIE1 and WT HCMV. Several genes were strongly upregulated after ΔIE1-infection, indicating that IE1 negatively affects the expression of these genes during HCMV infection. Significant upregulations could be observed for the genes *TNFSF10* (3.8-fold change to WT infection), *TNFRSF14* (3.2-fold) and *MYD88* (1.9-fold). A comparison of these data with a recent RNA sequencing experiment in IFN-β stimulated HFF revealed that these top hits are interferon-stimulated genes (Figure 7, ISGs depicted in pink) [48]. These results support our hypothesis that IE1 prevents necroptotic cell death during HCMV infection via inhibition of IFN-stimulated gene expression. In summary, our data strongly suggests that IE1 plays a pivotal role for the antagonism of necroptotic cell death, not only by a direct interaction with the necroptotic cascade, but also by modulating innate immune signaling.

## 4. Discussion

Human cytomegalovirus (HCMV) has been found to act as master manipulator of intrinsic as well as innate immune mechanisms. The immediate-early 1 protein (IE1) acts as protagonist in several of these strategies, e.g., due its activity on PML-NBs and the antagonism of IFN-signaling [30,32]. In 2015, Omoto et al. suggested a role of IE1 or an IE1-regulated gene product as antagonist of necroptotic cell death, but the mechanism remained unclear [24]. In this study, we further analyzed necroptotic signaling during HCMV infection and identified IE1 itself to exhibit strong anti-necroptotic activities.

First of all, we established a necroptosis-sensitive cell culture model with HCMV permissive cell lines. Via lentiviral transduction, we restored the expression of the necroptosis-initiating protein RIPK3 in fibroblasts (HFF) and endothelial cells (HEC-LTT). By using a cell viability assay, we showed that the reintroduction of RIPK3 confers the sensitivity to necroptosis, as without RIPK3 expression no significant cell death was induced (Figure 1C). The importance of RIPK3 expression for the execution of necroptosis is reported in several studies, demonstrating that RIPK3 deficiencies are linked to limited necroptosis induction [57,58]. As HCMV has been described to antagonize necroptotic cell death, we expected a modulation of the necrosome complex during infection. This expectation was fulfilled as we observed a massive upregulation of the post-translational modifications (PTMs) of the necrosome components RIPK1 and RIPK3 in Western blot analysis (Figure 2). This modulation can be observed after infection with two HCMV strains (AD169 and TB40/E) and in both cell types (HFF and HEC-LTT). Intriguingly, TB40/E infection resulted in a sharp upregulation of PTMs at 18 hpi in HFF, whereas for AD169-infected cells, a gradually increasing modification over time was observed. Based on this result, we hypothesized that the counteraction of necroptotic cell death is more efficient during TB40/E infection. Of note, we observed a rapid decrease in IE1 levels in HEC-LTT cells. The loss of IE1 expression during infection in HEC-LTT was also detected in our previous studies using this cell line and might be explained by an altered transcriptional regulation or an altered stability of IE1 in HEC-LTT cells compared to HFF cells [39].

The hypothesis that necroptosis is more efficiently counteracted during TB40/E than AD169 infection was confirmed by comparing these HCMV strains in a cell viability assay (Figure 3A). Both HCMV strains were found to exert a strong inhibition of TNF-induced necroptosis. For AD169-infected cells, a cell viability of approx. 65% was detected, whereas for TB40/E-infected cells a cell viability of approx. 83% was reached. This observation goes in line with the findings of Omoto et al. (2015) who had compared several HCMV lab strains, including AD169 and TB40/E, in their counteraction of necroptotic cell death [24]. The more efficient counteraction of necroptosis by TB40/E is most likely attributed to the activity of the HCMV protein pUL36. pUL36 has already been described as a potent inhibitor of apoptosis and is also termed as viral inhibitor of caspase-8 activation (vICA) [59]. Recently, pUL36 was found to exert an additional anti-necroptotic activity by targeting the necroptosis-executing protein MLKL for proteasomal degradation [25]. For the anti-apoptotic as well as for the anti-necroptotic activity of pUL36, residue 131 was proposed to be of high importance [25,59]. Intriguingly, sequence alignment revealed that AD169 exhibits a mutation at this position (arginine—R), compared to TB40/E (cysteine—C) and other HCMV strains like Toledo or Merlin [59]. This mutation in AD169 was shown to abrogate the anti-apoptotic and anti-necroptotic activity of pUL36 [25,59]).

Despite expressing a pUL36 that is incapable of degrading MLKL, AD169 is able to effectively counteract necroptosis at high levels. This suggests either the presence of another strong antagonist of necroptotic cell death or an additional anti-necroptotic activity of pUL36 that has not been described so far. By performing infection kinetics and monitoring cell viability in parallel, we could demonstrate that the antagonizing components are expressed at early times of infection (Figure 3B). By using an IE1-deleted AD169 strain, we could confirm the hypothesis of Omoto et al. that IE1 plays a critical role in the antagonism of necroptotic cell death, since a less efficient counteraction of necroptotic cell death was observed [24]. The use of UV-inactivated viruses in our studies showed that tegument proteins are most likely not involved in the antagonism of necroptosis at the early stages of infection, further emphasizing the role of IE1 in this process. Based on this data, however, we cannot rule out an additional activity of pUL36 beyond its degrading activity on MLKL.

As the presence of IE1 is crucial for the expression of other viral proteins and the progress of viral infection [26], it was essential to validate our data in an additional complementary approach. By using HFF/RIPK3 cells with inducible IE1 expression, we confirmed our data regarding IE1’s anti-necroptotic activity. The IE1 expressing cells showed a strong counteraction of necroptotic cell death by maintaining the cell viability on high levels of approx. 90% after TBZ treatment (Figure 4A). Thus, our results suggest the role of IE1 as a key antagonist of necroptosis during HCMV infection, and pUL36 possibly functions as an additive antagonist. 

Concerning the mechanism of necroptosis inhibition, we report that IE1 directly interferes with necroptotic signaling at the early stages by interacting with RIPK3 in an RHIM-independent manner (Figure 5). This finding was striking, as for herpesviruses predominantly RHIM-domain-dependent interactions with the necrosome were described [20,21]. So far, only human gammaherpesvirus 4 (HHV-4/EBV) was suggested to utilize a similar strategy [52]. Furthermore, the use of truncated proteins revealed that the disordered C-terminus of IE1 promotes the interaction with RIPK3. Since the binding site for STAT proteins, which is essential for inhibition of IFN signaling is located in the C-terminal region as well, further IE1 mutants with an altered C-terminus could help to dissect the contribution of RIPK3 binding and STAT binding to the inhibition of necroptosis. As a consequence of the interaction of IE1 and RIPK3, IE1 appears to manipulate the modification pattern of RIPK3, in particular, the ubiquitination of RIPK3 (Figure 4B–E). Interestingly, the promotion of RIPK3 ubiquitination and the interaction with RIPK3 appeared to depend on the C-terminal domain of IE1 (Figure 5E and Figure S2). This suggests that the C-terminal domain of IE1 plays a crucial role, on one hand, in the ubiquitination of RIPK3, and on the other hand, for the interaction of RIPK3 and IE1.

For RIPK3, several sites of ubiquitination are described some of which are associated with an attenuated execution of necroptosis [18,19]. In particular, so far the most prominent ubiquitination of RIPK3 is the K48-linked ubiquitination, which typically initiates the degradation of the linked protein [60]. So far, we could not observe any degrading effect of IE1 on RIPK3; therefore, we rather propose an alternative mechanism. IE1 might induce a ubiquitination that is linked to a non-degradative role such as altering the trafficking or the localization of a protein, e.g., the K63-linked ubiquitination [61]. There is also evidence that IE1 acts as an E3 ubiquitin ligase itself, as this activity is suggested by Liu and colleagues [62]. Whereas IE1 is known to mainly localize within the nucleus, RIPK3 is described to perform a constant shuttling between the nucleus and cytoplasm during the execution of necroptosis [63]. An altered trafficking of RIPK3 due to ubiquitination by IE1, could lead to an accumulation of RIPK3 within the nucleus and could therefore attenuate necroptosis. Employing RIPK3 ubiquitination mutants and immunofluorescence or live cell imaging approaches could provide further valuable insights. Interestingly, the anti-necroptotic strategy of human gammaherpesvirus 4 (HHV-4/EBV), which relies likewise on an RHIM-independent interaction with the necrosome, is based on a modulation of the ubiquitination pattern of RIPK3 and RIPK1 [52]. Alternatively, IE1 could affect the recruitment of interaction partners to RIPK3 to limit the execution of necroptosis. DNA sensing pathways display an important role in innate immunity and the DNA sensor ZBP1 (Z-nucleic acid binding protein 1, also known as DAI) was reported to play a critical role in the initiation of necroptosis. ZBP1 is found to interact with RIPK3 and thereby promotes the execution of necroptosis during infection with HSV-1, MCMV and influenza A virus [64,65,66]. By manipulating the PTMs of RIPK3, IE1 might prevent the interaction of ZBP1 and RIPK3, consequently leading to the abrogation of necroptosis induction by ZBP1.

Besides the direct anti-necroptotic activity of IE1, we also suggest an indirect activity of IE1 by antagonizing the interferon (IFN)-mediated upregulation of the necroptosis executioner MLKL (Figure 6). In general, MLKL has been reported to be upregulated upon IFN stimulation for type I and II IFNs in cancer cells [53,67], but so far, no type I IFN–mediated upregulation of MLKL in human fibroblasts was reported. Interestingly, the IFN-mediated upregulation of MLKL was reported to be due to the repression of a specific microRNA (miRNA). The miRNA miR-324-5p was found to repress the expression of MLKL under normal conditions, but upon IFN-signaling this repression is relieved [68]. This finding further emphasizes the close interplay between IFN-signaling and the execution of necroptotic cell death. Besides MLKL, we identified several components of innate immune signaling pathways to be antagonized by IE1 during infection by using necroptosis-related profiling (Figure 7). We propose that the global modulation of innate immune signaling by IE1 generates a beneficial cellular environment for HCMV, which facilitates productive replication. To further characterize the identified factors concerning their importance for the execution of necroptosis, a knockdown approach using specific siRNAs could provide additional insights in the necroptosis pathways.

## 5. Conclusions

In summary, our results show that IE1 plays a pivotal role in antagonizing necroptosis during HCMV infection. Several mechanisms, acting either directly or indirectly, were found to contribute to the anti-necroptotic effects of IE1. We detected a direct interaction of IE1 with RIPK3, leading to alterations in the PTMs of this important necrosome component. Additionally, IE1 indirectly influences necroptotic signaling via antagonizing IFN-mediated upregulation of the necroptosis executioner MLKL and several components of the innate immune response in order to establish a favorable environment for viral dissemination.

## Data Availability

Data are contained within the article and Supplementary Material.

## Supplementary Materials

The following supporting information can be downloaded at: https://www.mdpi.com/article/10.3390/v16020290/s1, Table S1: sequences of oligonucleotides used for cloning and RT-qPCR analysis; Figure S1: UV-inactivation of AD169; Figure S2: the ubiquitination of RIPK3 is not promoted by the IE1 mutant IE1 aa1-382.
