# Supplementary material for: IE1 of Human Cytomegalovirus Inhibits Necroptotic Cell Death via Direct and Indirect Modulation of the Necrosome Complex
Source: Viruses. 2024 Feb 13;16(2):290. doi: 10.3390/v16020290 (PMC10893529; doi:10.3390/v16020290)
Supplement: Supplementary file 1 [file viruses-16-00290-s001.zip › viruses-2814429-supplementary.pdf]

Table S1: Sequences of oligonucleotides used for cloning and RT-qPCR analysis

**Oligonucleotides for generation of FLAG-RIPK3 (pHM4730)**
5′CATATTAATTAAATGGACTACAAAGACGATGACGACAAGTCGTGCGTCAAGTTATGGC<br>3′CATAGTCGACTTATTTCCCGCTATGATTATAC
**Oligonucleotides for generation of RIPK3-MYC (pHM5136)**
5′CATAAAGCTTATGTCGTGCGTCAAGTTAT<br>3′CATACTCGAGTTACAGATCCTCTTCTGAGATGAGTTTTTGTTCTTTCCCGCTATGATTATAC
**Oligonucleotides for generation of FLAG-RIPK3 aa1-518 (pHM5304)**
5′CATAATCGATATGTCGTGCGTCAAGTTATG<br>3'CATACTCGAGTTATTTCCCGCTATGATTATACC
**Oligonucleotides for generation of FLAG-RIPK3 aa1-476 (pHM5305)**
5′CATAATCGATATGTCGTGCGTCAAGTTATG<br>3′CATACTCGAGTTAGTTGGGCAAGGCAGTTGT
**Oligonucleotides for generation of FLAG-RIPK3 aa1-443 (pHM5306)**
5′CATAATCGATATGTCGTGCGTCAAGTTATG<br>3′ CATACTCGAGTTATGGATTTGGCTCCGGGG
**Oligonucleotides for generation of FLAG-RIPK3 aa1-355 (pHM5324)**
5′CATAATCGATATGTCGTGCGTCAAGTTATG<br>3′CATACTCGAGTTACTCTAGATTCAGTTTGTTTAGCCA
**Oligonucleotides for generation of FLAG-MLKL (pHM5117)**
5′CATATTAATTAAATGGACTACAAAGACGATGACGACAAGGAAAATTTGAAGCATATTATCAC<br>3′CTAGTCTAGATTACTTAGAAAAGGTGGAGAGTT
**Oligonucleotides for generation of FLAG-IE1 FL (pHM5156)**
5′CATAGGATCCATGGAGTCCTCTGCCAAGAG<br>3′CTAG TCTAGA TTA CTGGTCAGCCTTGCTTC
**Oligonucleotides for generation of FLAG-IE1 aa1-382 (pHM5153)**
5′CATAGGATCCATGGAGTCCTCTGCCAAGAG<br>3′CTAG TCTAGA TTA CTCTTCCTCATCTGACTCCT
**Oligonucleotides for detection of *MLKL* in RT**
5′TTCACCCATAAGCCAAGGAG<br>3′GGATCTCCTGCATGCATTTT
**Oligonucleotides for generation of PCR product for generation of HCMV ΔIE1 strains**
57-75: 5′<br>AAAGATGTCCTGGCAGAACTCGGTAAGTCTGTTGACATGTATGTGATATAAGGATGACGACGATAAGTA<br>GGG

57-76: 3′<br>TAGTTTACTGGTCAGCCTTGCTTCTAGTCACCATAGGGTGGGTGCTCTTGTATATCACATACATGTCAACA<br>GACTTACCGAGTTCTGCCAGGACATCTTTCAACCAATTAACCAATTCTGATTAG<br>57-77: 5′ AGGATGACGACGATAAGTAGGG<br>57-78: 3′ TAGTTTACTGGTCAGCCTTG
**Oligonucleotides for PCR after second recombination and sequencing of HCMV ΔIE1 strains**
5′ ATGGAGTCCTCTGCCAAGAGA<br>3′ GCGTGACACGTTTATTGAGTAGG

Supplementary Figures S1 and S2

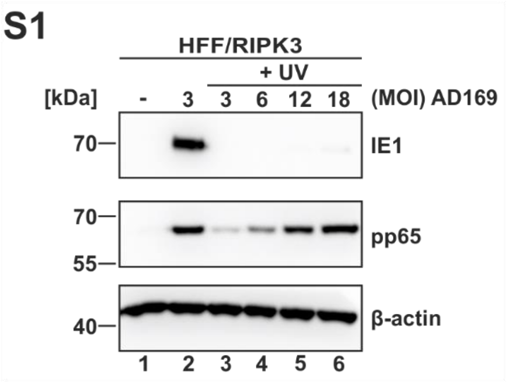

Figure S1: UV-inactivation of AD169. HFF/RIPK3 cells were infected with AD169 and UV-inactivated AD169 at MOIs 3 - 18 (UV-inactivation was conducted for 2 min with 0.12 J/cm²). Expression levels of IE1 and pp65 were monitored in Western blot analysis. β-actin served as internal loading control. The experiment was performed two times and one representative experiment is shown

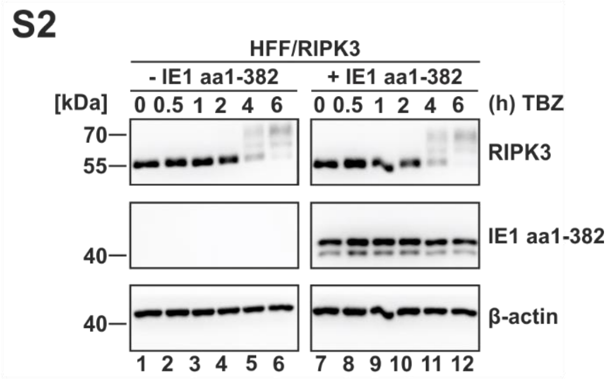

Figure S2: The ubiquitination of RIPK3 is not promoted by the IE1 mutant IE1 aa1-382. HFF/RIPK3 cells with inducible IE1 aa1-382 expres-sion were treated with TBZ for indicated times and subsequently the levels of RIPK3 and MLKL were monitored in Western blot analysis. - IE1 aa1-382, no IE1 aa1-382 expression, + IE1 aa1-382, IE1 aa1-382 expression. The experiment was performed two times and one representative experiment is shown.
